# Risk of infantile atopic dermatitis in neonatal lupus erythematosus: a retrospective cohort study

**DOI:** 10.3389/fimmu.2025.1517687

**Published:** 2025-03-27

**Authors:** Wenqiang Sun, Yihui Li, Xinyun Jin, Huiwen Li, Zexi Sun, Huawei Wang, Xue Liu, Lili Li, Jinhui Hu, Jie Huo, Xueping Zhu

**Affiliations:** ^1^ Department of Neonatology, Children’s Hospital of Soochow University, Suzhou, China; ^2^ Department of Nephrology, Children’s Hospital of Soochow University, Suzhou, China; ^3^ Department of Neonatology, The Affiliated Suzhou Hospital of Nanjing Medical University, Suzhou, China; ^4^ Department of Neonatology, Huai’an Maternal and Child Health Hospital, Huaian, China; ^5^ Department of Neonatology, Yangzhou Maternal and Child Health Hospital, Yangzhou, China

**Keywords:** lupus erythematosus, neonate, atopic dermatitis, autoantibody, glucocorticoid, probiotics

## Abstract

**Objectives:**

The onset and progression of atopic dermatitis (AD) are closely linked to autoimmune status. While AD has been observed in children with neonatal lupus erythematosus (NLE), its relationship with perinatal factors remains unclear. This study aimed to identify early-life risk factors for the development of AD in children with NLE within their first two years of life.

**Methods:**

We conducted a multicenter, retrospective cohort study using electronic medical records and follow-up data from patients in the NLE cohort. Children were categorized into AD and non-AD groups based on whether they developed AD by age two. Univariate and multivariate analyses were performed to compare general and clinical data between the two groups.

**Results:**

AD incidence in NLE patients was 27.27 (21/77). Compared to the non-AD group, the AD group had significantly lower use of oral probiotics and intravenous gamma globulin, but higher rates of small-for-gestational-age (SGA) status, hypocomplementemia, thrombocytopenia, anti-SSA, anti-SSB, double antibody (anti-SSA, anti-SSB) positivity, antibiotic use, and systemic glucocorticoid (GC) treatment. Logistic regression analysis revealed that oral probiotics were a protective factor against AD, while double antibody positivity and systemic GC were risk factors.

**Conclusion:**

In children with NLE, oral probiotics were associated with a reduced risk of AD, while double antibody positivity and systemic GC administration significantly increased the risk of AD within the first two years of life. However, the limited sample size in this study warrants further findings.

## Graphical Abstract

Exogenous probiotic supplementation early in life serves as a protective factor, while double antibody positivity (anti-SSA/SSB) and the systemic use of glucocorticoids increase the risk of developing atopic dermatitis in children with neonatal lupus erythematosus.

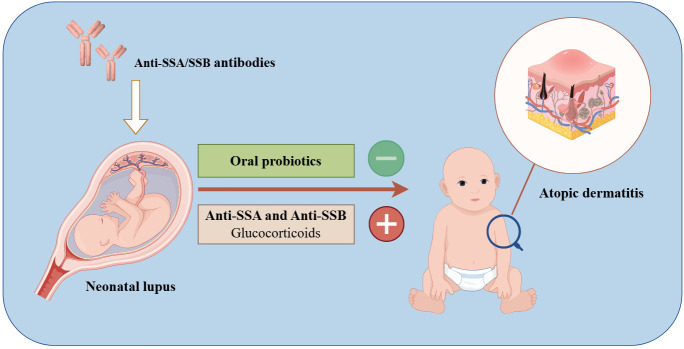

## 1 Introduction

Neonatal lupus erythematosus (NLE) is an acquired autoimmune condition caused by the transplacental transfer of maternal immunoglobulin G (primarily anti-SS-A and anti-SS-B antibodies) into the fetal circulation. This transfer targets fetal autoantigens, resulting in transient multiorgan involvement in the fetus (1, 2). Clinical symptoms of NLE typically appear at birth or within 4–6 weeks of life and may affect multiple organs, with skin manifestations being the most common, followed by hematological, hepatobiliary, and cardiac involvement (3, 4). These symptoms usually resolve within 6–12 months as maternal antibodies wane (4).

Atopic dermatitis (AD), or atopic eczema, is a chronic, recurrent inflammatory skin disease with a genetic predisposition, affecting 15–30% of children worldwide (5). Characterized by persistent itching and polymorphic skin lesions, AD is often the first stage in the “allergy march” and significantly impacts a child’s physical and mental development. The recurrent nature of AD leads to sleep disturbances and reduced quality of life, making it a particularly challenging condition to manage in childhood (6, 7).

During clinical studies and follow-up of patients with NLE, we observed a significantly higher rate of late-onset AD compared to its normal prevalence. While the pathogenesis of NLE and AD differs, autoimmune diseases are primarily mediated by Th1 cells, and allergic diseases are mediated by Th2 (8, 9). However, both are now understood to result from complex interactions between genetic, environmental, and other unknown factors (10). Recent evidence suggests a bidirectional relationship, with patients who have allergic diseases being at increased risk for autoimmune conditions (11, 12). Based on this, we hypothesized that certain clinical variables in patients with NLE may contribute to an elevated risk of developing AD. This study aimed to identify early-life risk factors for the development of AD in children with NLE within their first two years of life.

## 2 Material and methods

### 2.1 Study design and ethics approval

This is a multicenter retrospective cohort study analyzing early-life risk factors for AD development in patients with NLE. Patients with NLE hospitalized between January 1, 2011, and January 1, 2021, at the Children’s Hospital of Soochow University, the Affiliated Suzhou Hospital of Nanjing Medical University, Yangzhou Maternal and Child Health Hospital, and Huai’an Maternal and Child Health Hospital were included as study participants. Data were collected by reviewing electronic medical records from both inpatient and outpatient visits, supplemented by telephone and outpatient follow-up visits. The study was approved by the Ethics Committee of all Hospital (No. 2023CS024). Written informed consent was obtained from the guardians of all patients.

### 2.2 Study outcome

The primary outcome was whether NLE patients were diagnosed with AD before the age of 2. Clinical data and diagnostic information for all patients were reviewed and evaluated by specialized allergists to ensure diagnostic accuracy.

### 2.3 Diagnosis and definitions

The diagnosis of NLE was based on positive serum anti-SSA/SSB/U1RNP antibodies in pregnant women with autoimmune diseases or neonates with clinical manifestations of NLE (13). AD was diagnosed with reference to the Williams Clinical Diagnostic Criteria (14). Oral probiotics were counted from postpartum to 3 months after birth.

### 2.4 Exclusion criteria

Patients were excluded if they had significant deficiencies in clinical data that could introduce substantial bias, if their families declined participation, or if they had confirmed genetic defects or inherited metabolic diseases.

### 2.5 Data collection

Data were obtained from electronic medical records, outpatient clinics, and telephone follow-ups. Collected information included maternal history of rheumatologic diseases, parental history of allergic conditions, demographic characteristics, clinical presentations, laboratory and imaging results, and follow-up data. Demographic data included sex, gestational age (GA), and birth weight (BW). Laboratory investigations included routine blood counts, biochemical tests, and rheumatology-related serological tests. Imaging tests included ultrasonography, echocardiography, computed tomography (CT), and magnetic resonance imaging (MRI).

### 2.6 Statistical analysis

Statistical analyses were performed using SPSS version 26.0. Categorical data are presented as n (%) and were compared using chi-square or Fisher’s exact tests. Continuous data, which were non-normally distributed continuous data, are presented as medians with interquartile ranges (P25, P75) and analyzed using non-parametric tests. Logistic regression analysis was conducted with the occurrence of AD as the dependent variable, using significant indicators as independent variables. A *P*-value of < 0.05 was considered statistically significant.

## 3 Results

### 3.1 Clinical baseline characteristics

**Figure 1:**
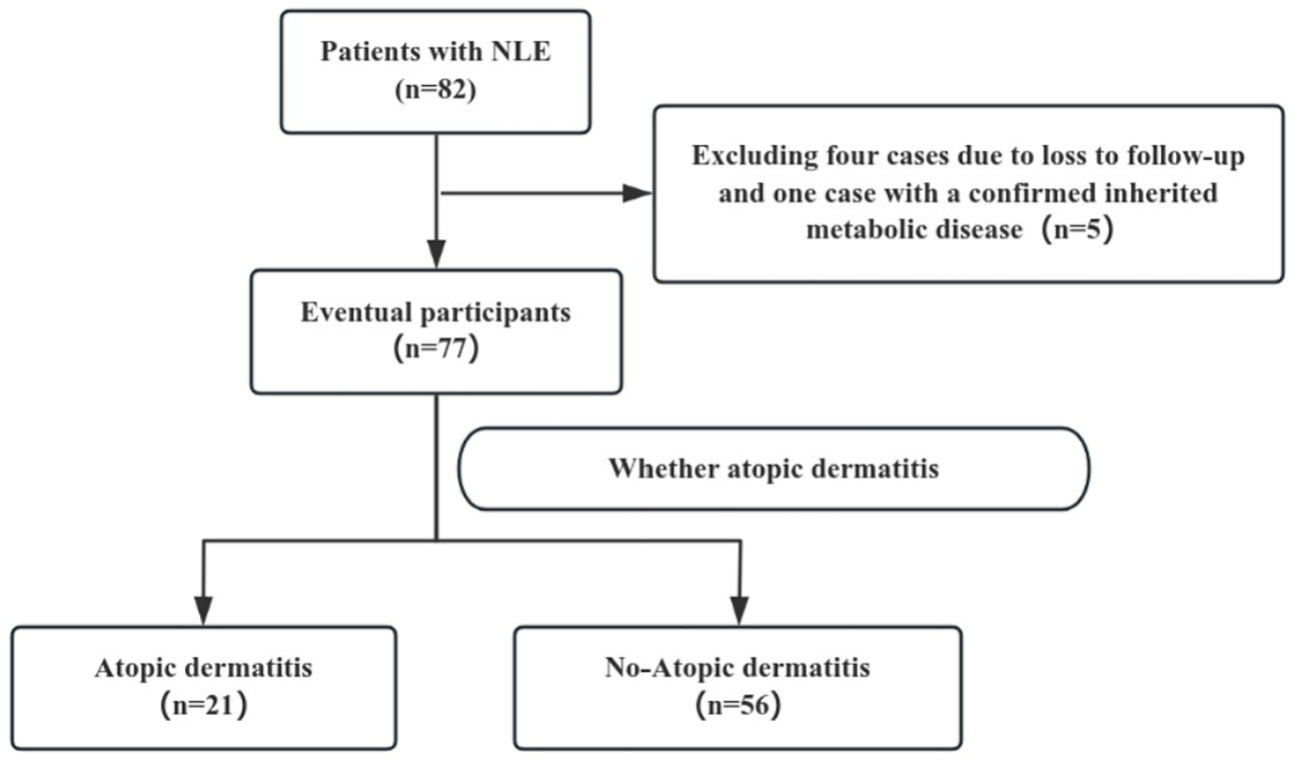
Flow chart of the study population. NLE, neonatal lupus erythematosus.

A flowchart of the study is provided in **Figure 1**. A total of 82 patients with NLE were hospitalized between January 1, 2011, and January 1, 2021. After excluding four cases due to loss to follow-up and one case with a confirmed inherited metabolic disease, 77 patients were finally included in the analysis. The cohort consisted of 31 males and 46 females, with a mean GA of 37^+2^ (35^+4^, 37^+6^) weeks, and a mean birth weight of 2425 (1795–2910) g. Among the patients, 38 had a GA ≥37 weeks, 28 had a GA between 32 and 37 weeks, and 11 had a GA <32 weeks. In terms of birth weights (BW), 38 patients weighed >2500 g, 27 weighed between 1500–2500 g, and 12 weighed <1500 g. Thirteen cases were younger than their gestational age. There were 34 cesarean deliveries and 43 vaginal deliveries. A history of allergic diseases was reported in 20 mothers and 15 fathers. Additionally, 47 patients had a history of oral probiotic, and 24 were breast feeding. By the age of two, 21 out of 77 patients with NLE were diagnosed with AD. See **Table 1**.

**Table 1:** Clinical baseline characteristics (n, %).

		AD (n=21)	No-AD (n=56)	*P-value*	NLE (n=77)
Sex	Male	9 (42.86)	22 (39.29)	0.776	31 (40.26)
	Female	12 (57.14)	34 (60.71)		46 (59.74)
Gestational age					
Term infant	≥37 weeks	7 (33.33)	31 (55.36)	0.085	38 (49.35)
Premature infant	<37 weeks, ≥32 weeks	12 (57.14)	16 (28.57)	0.020	28 (36.36)
Extremely preterm infant	<32 weeks	2 (9.52)	9 (16.07)	0.465	11 (14.29)
Birth weight					
Normal birth weight	>2500 g	8 (38.10)	30 (53.57)	0.642	38 (49.35)
Low birth weight	1500–2500 g	9 (42.86)	18 (32.14)	0.921	27 (35.06)
Very low birth weight	<1500 g	4 (19.05)	8 (14.29)	0.873	12 (15.58)
SGA		5 (23.81)	8 (14.29)	0.223	13 (16.88)
Mode of delivery	Cesarean section	9 (42.86)	25 (44.64)	0.888	34 (44.16)
	Vaginal delivery	12 (57.14)	31 (55.36)		43 (55.84)
Pet exposure during pregnancy		5 (23.81)	13 (23.21)	0.956	18 (23.38)
Mother allergic disease		6 (28.57)	14 (25.00)	0.750	20 (25.97)
Father allergic disease		4 (19.05)	11 (19.64)	0.953	15 (19.48)
Maternal autoimmune disease					
	SLE	11 (52.38)	32 (57.14)	0.708	43 (55.84)
	Photosensitivity symptoms	2 (9.52)	5 (8.93)	1.000	7 (9.09)
	MCTD	1 (4.76)	2 (3.57)	1.000	3 (3.90)
	Sjogren's syndrome	2 (9.52)	5 (8.93)	1.000	7 (9.09)
	Autoantibody abnormalities	2 (9.52)	4 (7.14)	1.000	6 (7.79)
	—	3 (14.29)	8 (14.29)	1.000	11 (14.29)
Oral probiotic		8 (38.09)	39 (69.64)	0.011	47 (61.04)
Days of oral probiotic		0 (17.50, 0)	14 (21, 0)	0.059	14 (21, 0)
Breast feeding		5 (23.81)	19 (33.93)	0.393	24 (31.17)

AD, atopic dermatitis; SGA, smaller than gestational age; SLE, systemic lupus erythematosus; MCTD, mixed Connective Tissue Disease.

There were no significant differences between the AD and non-AD groups regarding sex, BW, GA, extremely preterm birth, SGA status, mode of delivery, exposure to pets during pregnancy, parental history of allergic disease, maternal history of autoimmune disease, days of oral probiotic or breast feeding (*P*>0.05). Compared to the control group, the percentage of preterm births was significantly higher in the AD group, the percentage of oral probiotic in the neonatal period was significantly lower (*P*<0.05). See **Table 1**.

### 3.2 Clinical manifestations and laboratory tests

The percentage of patients with hypocomplementemia, thrombocytopenia, anti-SSA antibodies, anti-SSB antibodies, and double-positivity for anti-SSA and anti-SSB antibodies was significantly higher in the AD group compared to the non-AD group (*P*<0.05). However, no significant difference was observed between the two groups in terms of cutaneous manifestations (rash), anemia, neutropenia, coagulation abnormalities, congenital heart block, structural cardiac abnormalities, gastrointestinal involvement, or neurological involvement (*P*>0.05). Additionally, no significant differences were observed in anti-U1-RNP levels, antibody triple positivity (anti-SSA, Anti-SSB, Anti-U1-RNP), or serum eosinophil counts between the two groups (*P*>0.05). See **Table 2**.

**Table 2:** Clinical manifestations and laboratory tests (n, %).

		AD (n=21)	No-AD (n=56)	*P-value*	NLE (n=77)
Cutaneous	Total	18 (85.71)	47 (83.93)	0.977	65 (84.42)
Hematological	Total	16 (76.19)	36 (64.29)	0.368	52 (67.53)
	Anemia	9 (42.86)	28 (50.00)	0.530	37 (48.05)
	Hypocomplementemia	13 (61.90)	17 (30.36)	0.013	30 (38.96)
	Neutropenia/deficiency	6 (28.57)	18 (32.14)	0.727	24 (31.17)
	Thrombocytopenia	13 (61.90)	16 (28.57)	0.003	29 (37.66)
	Coagulation abnormalities	6 (28.57)	12 (21.43)	0.536	18 (23.38)
Cardiac	Total	12 (57.14)	26 (46.43)	0.442	38 (49.35)
	Congenital heart block	4 (19.05)	7 (12.50)	0.737	11 (14.29)
	Structural cardiac abnormalities	10 (47.62)	20 (35.71)	0.369	30 (38.96)
Gastrointestinal	Total	11 (52.38)	39 (69.64)	0.978	50 (64.94)
Neurological	Total	7 (33.33)	15 (26.79)	0.602	22 (28.57)
Antibodies	Anti-SSA	19 (90.48)	36 (64.29)	0.029	55 (71.43)
	Anti-SSB	16 (76.19)	24 (42.86)	0.011	40 (51.95)
	U1-RNP	7 (33.33)	14 (25.00)	0.492	21 (27.27)
	Anti-SSA and Anti-SSB	13 (61.90)	18 (32.14)	0.021	31 (40.26)
	Anti-SSA, Anti-SSB, Anti-U1-RNP	3 (14.29)	6 (10.71)	0.992	9 (11.69)
Eosinophil count×10^9^/L, IQR)		0.20 (0.13, 0.31)	0.18 (12, 0.28)	0.321	

AD, atopic dermatitis.

### 3.3 Main treatment during hospitalization

Compared with patients in the non-AD group, those in the AD group had a significantly higher percentage of antibiotic and systemic GC applications and a lower percentage of intravenous (IV) immunoglobulin applications (*P*<0.05). There were no significant differences in platelet counts, suspended oligoerythrocytes, or virus-inactivated plasma transfusions between the two groups (*P*>0.05). See **Table 3**.

**Table 3:** Main treatment during hospitalization (n, %).

	AD (n=21)	No-AD (n=56)	*P-value*
Antibiotic	15 (71.43)	25 (44.64)	0.043
Platelet	5 (23.81)	8 (14.29)	0.338
Red blood cell	7 (33.33)	21 (37.50)	0.695
Virus-inactivated plasma	4 (19.05)	8 (14.29)	0.897
Intravenous immunoglobulin	4 (19.05)	24 (42.86)	0.047
Systemic application of GC	17 (80.95)	23 (41.07)	0.002

AD, atopic dermatitis; GC, glucocorticoids.

### 3.4 Independent risk factor analysis

Logistic regression analyses were performed with the occurrence of AD as the dependent variable, using indicators of significant differences between the AD and non-AD groups as independent variables. The results revealed that oral probiotics (OR: 0.235, 95%CI: 0.059–0.942) served as a protective factor against the development of AD within the first two years of life in patients with NLE. Conversely, double positivity for anti-SSA and anti-SSB antibodies (OR: 4.213, 95%CI: 1.034–17.165) and systemic GC application (OR: 4.408, 95% CI: 1.248–15.568) were identified as risk factors. See **Table 4**.

**Table 4:** Independent risk factor analysis.

	*P-value*	HR(95%CI)
Oral probiotics	0.041	0.235 (0.059-0.942)
Hypocomplementemia	0.380	0.561 (0.154-2.041)
Thrombocytopenia	0.557	0.629 (0.134-2.958)
Anti-SSA	0.125	4.183 (0.673-26.010)
Anti-SSB	0.289	1.890 (0.582-6.135)
Anti-SSA and Anti-SSB	0.045	4.213 (1.034-17.165)
Antibiotic	0.863	1.186 (0.171-8.221)
Intravenous immunoglobulin	0.078	0.341 (0.103-1.13)
Systemic application of GC	0.021	4.408 (1.248-15.568)

GC, glucocorticoids.

## 4 Discussion

The microecology of the human gastrointestinal tract plays an important role in the onset and progression of allergic conditions by influencing anti-allergic mechanisms such as Th1 immunity, TGF signaling, and IgA production (15). Early colonization and development of the gut microbiota are thought to be closely linked to the risk of allergies in infancy, childhood, and adulthood (16). A large number of studies have confirmed that prenatal and postnatal probiotic supplementation may be an effective means of preventing AD in children, but there is heterogeneity in the results of the existing studies, and further confirmation is still needed (17–19). In this study, the percentage of patients in the AD group who used oral probiotics was significantly lower than that in the non-AD group, while the use of intravenous antibiotics was significantly higher. In addition, among the children with NLE in this study, the number of days of oral administration of probiotics was higher in non-AD children than in children in the AD group, although there was no statistical difference between the two groups, which may be related to the smaller sample size. Although the study population was limited to patients with NLE, our findings suggest that early probiotic supplementation may help maintain a balanced gut microbiota, potentially serving as a therapeutic strategy to prevent the development of allergic diseases.

Relevant studies have shown that anti-SSA/SSB/U1RNP antibodies are associated with rashes in children with NLE, with double positivity for anti-SSA and anti-SSB, as well as triple positivity (Anti-SSA/SSB/U1RNP antibody), greatly increasing the incidence of rashes in patients with NLE (20). Histological examinations have revealed granular IgG deposits at the dermal-epidermal junction and vesicular changes at the skin’s interface and adnexal structures (21). Additionally, many studies have confirmed that immune complex deposition plays an important role in the development of NLE rash. However, to our knowledge, no studies have directly linked autoimmune antibodies to the development of AD. In our study, we found that children with NLE who developed AD had significantly higher rates of anti-SSA, anti-SSB, and anti-SSA/SSB double antibody positivity compared to the non-AD group, identifying double antibody positivity as a risk factor for AD. This may be due to NLE-associated antibodies on processes such as apoptosis, inflammatory responses, T-cell activation and proliferation, and pro-inflammatory interleukin production—mechanisms that are also central to the pathogenesis of AD (22–24).

The use of GC in patients with NLE is common, with intravenous GC administered to 40 patients in our study. The rate of IV GC use was significantly higher in the AD group compared to the non-AD group, identifying it as a risk factor for the development of AD in patients with NLE by age 2. While GCs are typically used to regulate skin homeostasis and are the first-line topical treatment for AD (25, 26), studies on the impact of early-life intravenous GC administration on AD risk are limited. Fetuses and newborns are particularly sensitive to GC, and therapeutic doses have lasting effects on growth, organ function, and immune function (27, 28). Early exposure to GC may increase immune reactivity to foreign antigens and compromise the skin’s natural barrier, both of which increase the risk of allergic diseases.

Epidemiological studies have consistently shown that children exposed to antibiotics in the first months or years of life have a significantly increased risk of developing AD and asthma (16, 29–31). The use of antibiotics early in life disrupts the normal evolution and colonization of gut microorganisms, which, in turn, alters the host’s immune status, thus increasing the susceptibility to AD (16). This was corroborated by the significantly higher percentage of patients in the non-AD group who were taking oral probiotics. In this study, the percentage of antibiotic use in patients in the AD group was significantly higher than that in the non-AD group; however, after multifactorial regression analysis, it was not found to be an independent risk factor for AD development in patients with NLE. Most of these studies, including ours, were observational, and patients on antibiotics often have bacterial infections and varying underlying immune statuses; therefore, more confounding factors may be involved.

This study is the first to establish a correlation between perinatal factors and the occurrence of AD within the first two years of life in patients with NLE. Although the study included patients from four clinical centers, the rarity of NLE resulted in a relatively small final sample size. To reduce bias, we utilized electronic case retrieval, along with outpatient and telephone follow-ups, ensuring all relevant information was collected and cross-checked by specialized physicians in pairs to. In addition, it is important to note that, based on the retrospective of this study, whether relevant factors, including oral probiotics in NLE patients, are associated with the development of AD needs to be further verified. Previous studies have shown that dupilumab or oral JAK inhibitors have good therapeutic effects and a high safety profile in patients with moderate-to-severe AD. Among them, dupilumab can significantly reduce related comorbidities in pediatric AD patients (32, 33). In the future, we need to conduct large-scale, multicenter prospective studies to further explore and confirm the long-term risk factors for developing AD in NLE patients, as well as the corresponding treatment strategies.

Our results indicate that early exogenous probiotic supplementation may be a protective factor against the development of AD in patients with NLE by age 2, while anti-SSA/SSB double positivity and intravenous GC use are risk factors. However, further research and prospective clinical trials are required to elucidate the causal relationships of these associations.

## Data Availability

The original contributions presented in the study are included in the article/Supplementary Material. Further inquiries can be directed to the corresponding author.
